# Investigation of *CYP1B1* mutations in Chinese patients with primary congenital glaucoma

**Published:** 2009-02-27

**Authors:** Mei Yang, Xiangming Guo, Xing Liu, Huangxuan Shen, Xiaoyun Jia, Xueshan Xiao, Shiqiang Li, Shaohua Fang, Qingjiong Zhang

**Affiliations:** State Key Laboratory of Ophthalmology, Zhongshan Ophthalmic Center, Sun Yat-Sen University, Guangzhou, People’s Republic of China

## Abstract

**Purpose:**

This study was conducted to investigate the mutation spectrum of the cytochrome P450 gene (*CYP1B1*) in Chinese patients with primary congenital glaucoma (PCG).

**Methods:**

The coding regions of *CYP1B1* from 41 Chinese PCG patients were analyzed using polymerase chain reaction (PCR) and heteroduplex analysis-single strand conformation polymorphism (HA-SSCP) followed by subsequent cloning and bidirectional sequencing. New variants were confirmed by restriction fragment length polymorphism (RFLP) analysis in 80 normal Chinese controls.

**Results:**

Six distinct mutations, four of which are novel, were identified in 14.6% (6/41) of all patients. The *CYP1B1* mutations in two patients were homozygous, and the other four patients were compound heterozygous. Beyond the four novel mutations (g.4531_4552del22bp, g.4633delC, p.S336Y, and p.I471S), two reported missense mutations (R469W and R390H) were also identified. The missense mutation, R390H, was involved in 9.8% (4/41) of patients in our study. None of the novel mutations was observed in any of the 80 controls.

**Conclusions:**

Our results support the premise that *CYP1B1* is a major gene for PCG, appearing to be responsible for the disease in roughly one in six Chinese PCG patients. The R390H mutation was identified as a predominant *CYP1B1* allele among the Chinese PCG patients in our study. This observation emphasizes the importance of mutational screening of *CYP1B1*, especially for the R390H mutation in Chinese patients.

## Introduction

A rare but severe primary congenital form of glaucoma (PCG; OMIM 231300), which usually manifests itself from birth to three years of age, is a significant cause of blindness in children [1,2]. In primary congenital glaucoma (PCG), the ocular developmental defects of the trabecular meshwork and anterior chamber angle lead to the obstruction of aqueous outflow and subsequent increased intraocular pressure (IOP). PCG is detected clinically in neonates and infants by noting the typical symptoms of photophobia and epiphora as well as signs of globe enlargement, edema and opacification of the cornea, and ruptures involving Descemet’s membrane, all of which result from the elevated IOP [3].

Even though three different chromosomal loci for PCG on 2p21 (glaucoma 3, primary congenital, A [GLC3A, OMIM 231300]) [4], 1p36 (GLC3B) [5], and 14q24.3 (GLC3C) [6] have been mapped, the cytochrome P450 gene, *CYP1B1* (OMIM 601771), located in the GLC3A locus, has been characterized. The human *CYP1B1* consists of three exons with the translated region beginning at the 5′ end of the second exon. It encodes a member of the cytochrome P450 superfamily, subfamily I. The *CYP1B1* product is a 543 amino acid long protein that contains conserved core structures (CCS), which includes four helix bundles (helices D, I, and L and the antiparallel helix E), helices J and K, β-sheets 1 and 2, a heme-binding region, and a “meander” region. Most of these regions are located within the COOH-terminal half of the CYP1B1 protein and are expected to be involved in heme-binding and proper folding of the molecule [7]. CYP1B1 is a membrane-bound monomeric mixed function mono-oxygenase that is expressed primarily in the trabecular meshwork but also in the posterior segment of the eye, notably in the neuroretina, and other tissues such as the adrenal gland, the ovary, the testis, the lung, the uterus and the kidney [8]. The protein is probably associated with the metabolism of compounds that are critical to the developing eye [9].

Mutations in *CYP1B1* is the predominant cause of autosomal recessive inherited PCG that has been reported in various ethnic backgrounds [6,10-13]. So far, the spectrum of *CYP1B1* mutations causing PCG in the Chinese population is not yet well understood. Herein, we investigated *CYP1B1* mutations in 41 unrelated Chinese patients with PCG.

## Methods

### Patient recruitment

The study followed the tenets of the Declaration of Helsinki with written informed consent obtained from all patients or from their parents if their age was less than 18. Forty-one patients with PCG were recruited from the glaucoma clinics at Zhongshan Ophthalmic Center at Sun Yat-sen University (Guangzhou, China). After undergoing a complete eye examination including slit-lamp biomicroscopy, optic nerve examination, and measurement of IOP by Perkins or Goldmann tonometry, all enrolled patients were diagnosed with PCG. Subjects with secondary causes of glaucoma (e.g., trauma, uveitis, steroid-induced- or neovascular glaucoma, and other associated ocular or systemic anomalies) were excluded. PCG was defined according to the following criteria: age of onset less than three years; IOP greater than 21 mmHg without any treatment; optic nerve cupping, and moreover, rupture of Descemet’s membrane, or horizontal corneal diameter greater than 12 mm with or without corneal edema. Patients older than three years without secondary causes but with buphthalmos were considered to have PCG.

In our study, six (14.6%) patients had unilateral PCG, and neither a family history of PCG nor parental consanguinity was established in any of the 41 unrelated Chinese patients. Eighty ethnically matched normal individuals without any ocular disorders served as controls.

### Mutation screening

Blood samples were collected from 41 patients and 80 controls after informed consent was received. Genomic DNA was extracted from leucocytes by proteinase K-phenol-chloroform extraction. Ten sets of primers (Table 1) were used to amplify the coding regions of *CYP1B1* (U56438). Polymerase chain reaction (PCR) amplification was performed in a 25 l volume by means of a touchdown PCR program with an annealing temperature decreasing from 64 °C to 58 °C or 56 °C over 10 cycles followed by 30 cycles with an annealing temperature of 58 °C or 56 °C. Subsequently, the PCR products were analyzed by heteroduplex analysis-single strand conformation polymorphism (HA-SSCP) as described previously [14]. PCR products showing aberrant banding patterns on SSCP were sequenced using the ABI BigDye Terminator Cycle Sequencing Kit v3.1 (Applied Biosystems, Foster City, CA) in accordance with the manufacturer’s recommendations, using an ABI 377 or 3100 sequencer (Applied Biosystems). The fragment containing the entire second exon was amplified for sequencing by the first set of forward primers and the sixth set of reverse primers (Table 1), the third exon amplified by the seventh set of forward primers and the tenth set of reverse primers (Table 1). Each variant was confirmed by bidirectional sequencing. Comparative sequencing analysis between different samples and the standard sequence (U56438) was performed using the SeqManII program of the Lasergene package (DNAStar Inc., Madison, WI). Heterozygous deletions (g.4531_4552del22bp and g.4633delC) were identified using a monoclone followed by direct sequencing.

**Table 1:** Primer pairs for *CYP1B1* mutation analysis.

**ID**	**Primer sequences (5′→3′)**	**Product length (bp)**	**Annealing temperature (°C)**
CYP1F	CTGAGTGTCACGCCTTCT	261	56
CYP1R	CGAGCGAACGAGAGGTGA		
CYP2F	CGTTTGCGTGGCCACTGAT	256	56
CYP2R	TCCGAGTAGTGGCCGAAAG		
CYP3F	CACGGCGCAGCGCAGATGC	242	56
CYP3R	GCCGAAACACACGGCACTCAT		
CYP4F	CCGTGGCCAACGTCATGAGT	256	56
CYP4R	GGCCGAAGGCTTTCGCAGT		
CYP5F	CGAGCAGCTCAACCGCAACT	183	56
CYP5R	GCCGGTACGTTCTCCAAATC		
CYP6F	AACGTACCGGCCACTATCAC	231	56
CYP6R	GACGCGATCTTGGTTTTGAG		
CYP7F	CACTGAGCTAGATAGCCTAT	253	58
CYP7R	GGAGAAGCGCATGGCTTCAT		
CYP8F	ATGGGTGACCAGCCCAACCT	238	58
CYP8R	GGCCGTCCTTGTCCAAGAAT		
CYP9F	TGGCCTAACCCGGAGAACTT	210	58
CYP9R	CATTTTCGCAGGCTCATTTGG		
CYP10F	GGCTCACCAGTGCGATTTCAG	228	58
CYP10R	ACTCCTCATCTCCGAAGATG		

Listed are primer sequences, sizes of PCR products, and the annealing temperature used for the amplification. Ten pairs of primers were used to amplify and sequence the *CYP1B1* genomic segments.

Four mutations were confirmed by restriction fragment length polymorphism (RFLP), I471S by BtsI, S336Y by AhdI, g.4531_4552del22bp by TfiI, and g.4633delC by SmlI. Digestion products were loaded onto an 8% (49:1) polyacrylamide/0.5X TBE gel and resolved at 18 W for about 2 h. Subsequently, the gel was silver stained as previously described, similar to how it was used in SSCP analysis [15]. The two missense mutations were analyzed by multiple amino acid sequence alignment of CYP1B1 and CYP1B2 from different species using the MegAlign program of the Lasergene package (DNAStar Inc.).

## Results

The complete coding region of *CYP1B1* from all 41 PCG patients was screened using HA-SSCP followed by direct sequencing. We identified six different mutations, four of which are novel, in six patients with bilateral PCG. We also identified five reported polymorphisms, g.3793 T>C, g.3947 C>G (R48G), g.4160 G>T (A119S), g.8131 C>G (L432V), and g.8184 C>T (D449D) [6]. Details of the six patients and mutations are summarized in Table 2.

**Table 2:** Summary of clinical findings and *CYP1B1* mutations in six PCG patients.

**Patient**	**Gender**	**Age at diagnosis**	**Mutation 1**	**Mutation 2**	**SNP**	**VA OD**	**VA OS**
G1	F	2 Y	g.8242C>T (R469W)	g.4633delC (p.F276FfsX1)	C/C	CF	LP
G13	M	3 Y	g.8006G>A (R390H)	g.4633delC (p.F276FfsX1)	C/C	CF	CF
G28	M	10 Mo	g.8006G>A (R390H)	g.4531del22bp (p.D242DfsX28)	C/C	LP	LP
G40	F	1 Y	g.8249T>G (I 471S)	g.8249T>G (I 471S)	C/C	LP	LP
G41	M	3 Y	g.8006G>A (R390H)	g.4812C>A (S336Y)	C/C	LP	0.2
G92	M	19 Y	g.8006G>A (R390H)	g.8006G>A (R390H)	C/C	CF	0.5–0.7

Clinical data, *CYP1B1* mutations, and single nucleotide polymorphisms (SNP, g.8131C>G) of the six PCG patients are summarized. GenBank U56438; “F”: female; “M”: male; “Y”: years; “Mo”: months; “SNP”: g.8183C>G(L432V); “LP”: light perception; “CF”: count fingers; “VA”: visual acuity.

### Analysis of the four novel mutations

**Figure 1:**
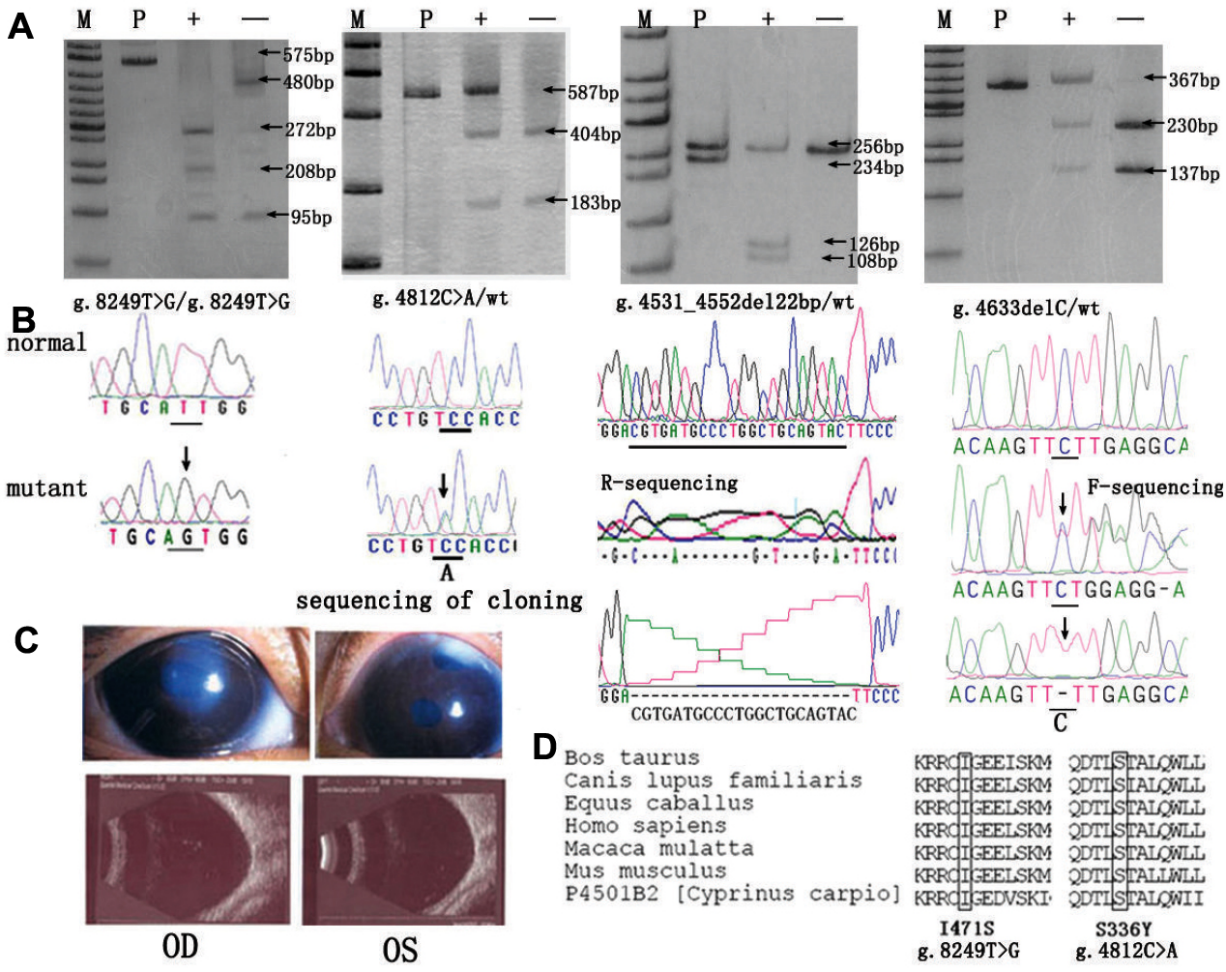
Four novel *CYP1B1* mutations observed in Chinese patients with PCG. **A**: Result of the restriction fragment length polymorphism (RFLP) analysis of the four mutations. “M”: marker; “P”: PCR patient products; “+”: restriction fragments from patients; “−”: restriction fragments from controls. The size of the fragments is indicated by an arrowhead. **B**: Sequencing of the four novel mutations and normal controls are shown. As for the deletions, sequencing of the cloning was shown. The exact mutation was labeled under each sequence according to the nomenclature recommended by HGVS. **C**: Pictures of the patient with I471S show the larger cornea and bigger eyeball. Photos of the anterior segment and B-mode ultrasonography of both eyes are also shown. **D**: Sequence alignment of seven different cytochrome P450 proteins revealed that the two novel missense mutations (S336Y, I471S) occurred at highly conserved positions.

The four novel mutations (Figure 1B) included two frame-shift mutations, both of them in the second exon, leading to deletions of 22 bp (g.4531_4552del22bp, reference sequence U56438) and 1 bp (g.4633del C), and two missense mutations (S336Y and I471S). Except for the homozygous I471S, each of the other three new mutations was in the compound heterozygous condition with another mutation. All four mutations changed one restriction site, g.4531_4552del22bp (TfiI) and I471S (BtsI) created new restriction sites, and g.4633delC (SmlI) and S336Y (AhdI) resulted in the loss of restriction sites. Using special restriction endonuclease digestion (Figure 1A), we failed to identify these four novel mutations in any of the 80 controls.

Because of the creation of premature stop codons, both deletions were predicted to truncate the *CYP1B1* open reading frame. The frameshift mutation, g.4531_4552del22bp (p.D242DfsX28), which affects the third nucleotide of codon 242, introduces a premature stop codon at codon 270, and g.4633delC (p.F276FfsX1) creates a premature stop codon at codon 277. Because the COOH-terminal half of the CYP1B1 protein is expected to be involved in heme-binding and proper folding of the molecule [7], the two deletions eliminating the essential COOH-terminal part of the P450 protein obviously affected the structure and damaged the function of CYP1B1.

Because the novel missense mutations altered hydrophobic properties at their sites (I471S) or consisted of replacement of the micromolecular Ser side chain of by the large hydrophilic Tyr (S336Y), the I471S and the S336Y could directly affect the helix L and the helix I of the conserved core structures (CCS) of CYP1B1 protein, respectively. These mutations could interfere with heme incorporation, by affecting the CCS that controls the proper folding and heme-binding ability of P450 molecules [7,16,17]. Comparative amino acid sequence alignment of seven different cytochrome P450 proteins revealed that the two novel missense mutations (S336Y and I471S) occurred at highly conserved positions (Figure 1D).

### Other mutations

Apart from these four novel mutations, two reported missense mutations were also found in our study. The R390H mutation previously reported in Pakistani and Indian patients [7,18] was the most frequent mutant allele identified in our study population. It exhibited heterozygosity or homozygosity in 9.8% (4/41) of the patients recruited. Another missense mutation, R469W, which has been reported in British and Turkish patients [7], was associated with the frameshift mutation (g.4633del C) in one patient in our study.

## Discussion

Because of ethnic differences and geographical variations, the prevalence of *CYP1B1* mutations varies in different patient populations from 100% in Saudi Arabians with PCG to 20% among the Japanese with PCG [6,10-13]. In our study, *CYP1B1* mutations were found in 6 out of our 41 Chinese PCG patients (14.6%).

Our study supports the contention that *CYP1B1* is a major gene involved in PCG, though the mutation rate in our study was less than the 20% level reported in sporadic Japanese PCG patients [6,10-13]. As for mutation screenings among different populations, the potential for bias (differences in the criterion for patient recruitment and the proportion of parental consanguinity among patients, etc.) must be considered. Populations who have a higher rate of consanguinity exhibit a higher frequency of *CYP1B1* mutations among PCG patients than ethnically diverse populations (like the Japanese and Chinese) [11,12]. The unevenness in the distribution of *CYP1B1* mutations among different populations may be due to founder effects [19]. Because *CYP1B1* mutations are responsible for only about 14.6% of Chinese PCG patients, it is imperative that other causes of PCG be investigated in future studies.

Although different *CYP1B1* mutation spectrums have been discovered in different populations with PCG, certain mutations occur repeatedly in ethnically distinct populations. Analysis of the constructed haplotype for *CYP1B1* was used to study these mutations [20-22]. If genetic markers on the mutant chromosomes are identical, the mutation is a founder mutation. However, if different genetic markers are observed, the recurrence of the mutations would be explained by a mutation ‘hot spot’. R368H, which is reported as a predominant *CYP1B1* allele in Indian PCG patients (17.8%) [20], and G61E, which is found in 72% of the Saudi-Arabians [8] and 47% of the Kuwaiti PCG patients [23], were identified as founder mutations in the Western world [22]. Because founder mutations are also the oldest mutations, they may be the most frequent mutations in PCG patients in ethnically heterogeneous populations.

The R390H mutation, which has been reported in 4.7% of Indian [19] and 3.6% of Pakistani [12] PCG patients, was the most frequent mutation observed in our study samples where 9.8% (4/41) of PCG patients were found to have this mutation. This is the highest reported frequency of this mutation of all ethnic backgrounds studied so far. As for the predominant mutation, R390H, found in our study, further studies will be needed to clarify whether this is the founder mutation in Chinese patients or just a mutation ‘hot spot’. Either way, screening for R390H may be a method for rapid detection of potential carriers and affected individuals.

The five reported polymorphisms, g.3793T>C, g.3947C>G (R48G), g.4160 G>T(A119S), g.8131C>G (L432V), and g.8184C>T (D449D), construct the 5′-CCGGTA-3′ haplotype associated with most *CYP1B1* mutations in the Western world [22]. The g.8131G (V432) polymorphism has the highest frequency in Brazilian PCG patients compared to normal controls [6]. Our results failed to show the association of the 5′-CCGGTA-3′ haplotype with *CYP1B1* mutations in Chinese patients, and g.8131G not g.8131C was found to be associated with all the patients with *CYP1B1* mutations (Table 2). The mutations identified in Chinese patients tend to be present on other haplotypes.

In summary, this study shows that 14.6% of Chinese patients studied carried *CYP1B1* mutations and that R390H appears to be the predominant mutant allele causing PCG in Chinese patients. In light of these findings, we suggest that screening for this mutation should be a priority for the scientific community.
